# Gastrointestinal symptoms and knowledge and practice of pilgrims regarding food and water safety during the 2019 Hajj mass gathering

**DOI:** 10.1186/s12889-021-11381-9

**Published:** 2021-07-01

**Authors:** Saber Yezli, Yara Yassin, Abdulaziz Mushi, Alhanouf Aburas, Lamis Alabdullatif, Mariyyah Alburayh, Anas Khan

**Affiliations:** 1 The Global Centre for Mass Gatherings Medicine, Public Health Directorate, Ministry of Health, Riyadh, Saudi Arabia; 2 Department of Emergency Medicine, College of Medicine, King Saud University, Riyadh, Saudi Arabia

**Keywords:** Hajj, Mass gathering, Health promotion, Public health, Gastrointestinal, Foodborne disease, Diarrhea

## Abstract

**Background:**

Food and water-borne diseases (FWBDs) are a health risk at the Hajj mass gathering. The current study documented the prevalence and management of gastrointestinal (GI) symptoms among pilgrims during the 2019 Hajj and assessed their knowledge and practice concerning food and water safety.

**Method:**

An analytical cross-sectional study was conducted in Macca, Saudi Arabia, among adult Hajj pilgrims from 28 countries. Data was collected from 15th–20th August 2019 by facer-to-face interviews using an anonymous structured questionnaire. Basic demographic data as well as information regarding pilgrims’ knowledge and practice relating to food and water safety and any GI symptoms experienced during the Hajj was collected and analyzed.

**Results:**

The study enrolled 1363 pilgrims with a mean age of 50.1 years (SD = 12.3) and 63.4% (*n* = 845) were male. At least 9.7% (*n* = 133) of pilgrims experience GI symptoms and 5.1% (69/1363) suffered diarrhea. Most respondents drunk bottled water (99.4%, *n* = 1324) and obtained their food from their hotel /Hajj mission (> 86%). In general, pilgrims had good knowledge and practice in relation to food and water safety, although risky practices were noted concerning keeping food at unsafe temperatures and hazardous sharing of food and water. Gender, nationality and suffering GI symptoms during Hajj were significantly associated with good knowledge and good practice. There was a moderate but statistically significant positive correlation between knowledge and practice scores (r_s_ = 0.41, *p* < 0.0001).

**Conclusion:**

Despite overall good knowledge and self-reported practice, risky behaviors relating to food and water safety were identified among pilgrims, many of whom suffered from GI symptoms during Hajj. Our results can form the basis for developing tailored, targeted and effective interventions to improve pilgrims’ knowledge and behavior and reduce the burden of FWBDs at the Hajj and beyond.

## Introduction

Food and water-borne diseases (FWBDs) are major global health issues with significant morbidity and mortality [1, 2]. Over 200 diseases are caused by ingesting food that is deteriorated and contaminated by microorganisms, such as bacteria, fungi, and viruses or parasites, as well as natural toxins, chemicals, and physical agents [3]. Contamination can occur at any stage of the food production, delivery and consumption chain. Although presenting mostly as gastrointestinal (GI) issues, foodborne diseases encompass a wide range of illnesses and may produce neurological, gynaecological, immunological and other symptoms [3]. Every year, nearly one in 10 people worldwide fall ill after eating contaminated food, leading to an estimated 420,000 deaths and 33 million healthy life years lost [2]. Half of these deaths are caused by diarrhoeal diseases, the most common illnesses resulting from the consumption of contaminated food. Similarly, consumption of unsafe and contaminated water is a major cause of illness and mortality resulting in an estimated 485,000 diarrhoeal deaths each year [1]. Ensuring food and water quality at sources, compliance with international and national standards and market regulations by producers as well as proper processing, cooking and storage by consumers are key to preventing FWBDs [3]. High level of knowledge and appropriate attitude and practice regarding food and water safety among food and water handlers and consumers reduces the risk of FWBDs. However, studies report that, in general, knowledge of food and water safety among these populations is inadequate and that there is no proper translation of knowledge/attitudes into practice [4–6].

Mass gatherings are congregated settings that favor the transmission of infectious agents including those responsible for FWBDs [7]. The increase in the number of persons in an area as the result of a mass gathering leads to a significant increase in the amount of food and water brought into the area, prepared, served and consumed as well as waste to be removed. As such, FWBDs and outbreaks are a public health risk at mass gatherings and food and water safety are a key consideration in the planning and management of such events [8]. The Hajj religious mass gathering in the Kingdom of Saudi Arabia (KSA) is annually attended by over 2 million pilgrims originating for over 180 countries. Pilgrims are housed in shared accommodations mainly in hotels pre and post Hajj and in purposefully built tents in the holy sites of Mina and Arafat during the Hajj rituals. FWBDs have been reported at the Hajj and the pilgrimage has been associated with numerous FWBDs outbreaks [9–11]. Several factors have been reported to contribute to this issue including: possible breaches of food hygiene standards, shortage of clean water, the presence of asymptomatic carriers of pathogenic agents, and the preparation of large numbers of meals that may be inappropriately stored by pilgrims [12].

KSA authorities have strict regulations to ensure the quality and safety of food and water at the Hajj. In addition, as a precautionary measure the Saudi authorities do not permit entry of food with arrivals for Hajj except in small quantities and properly canned or sealed containers [13]. The Saudi Ministry of Health also recommends that all pilgrims observe food and water safety measures such as hand washing, separation of raw and cooked food, thorough cooking of food and storing it at safe temperatures. However, it is unclear whether pilgrims follow these recommendations and their knowledge and practice regarding food and water safety at the Hajj are largely undocumented.

The study aimed to explore pilgrims’ knowledge and practice concerning food and water safety and highlight knowledge gaps or poor practices that may increase their risk of FWBDs. Also to investigate the prevalence and management of GI symptoms among the studied population.

## Method

### Study design setting and population

A analytical cross-sectional study, using a convenience sampling technique, was conducted among pilgrims in Macca, Saudi Arabia, during the 2019 Hajj season. The study was performed from the 15th–20th August 2019. The minimum sample size estimated for the study was 1067 based on a 3% margin of error, a confidence interval (CI) of 95%, an approximate pilgrims population of 2 million as well as an expected response proportion of 50% to most of the main questions. After adjusting for a projected 10% attrition, the estimated final sample size for the study was a least 1200 participants.

### Data collection tools and scoring system

Data was collected using an anonymous questionnaire administered via face-to-face interviews with pilgrims. Fifteen trained researchers with experience in performing studies during Hajj conducted the interviews and completed the questionnaires. Researchers spoke at least one of the five main languages spoken by pilgrims: English, Arabic, French, Malay or Urdu. In case pilgrims did not speak any of these languages, researchers coordinated with the pilgrims’ own medical or Hajj missions, who speak both English/Arabic and the pilgrims’ native tongue, to administer the questionnaire.

The study questionnaire was developed in Arabic and English and collected basic demographic data as well as information regarding pilgrims’ knowledge and practice relating to food and water safety and any GI symptoms experienced during the Hajj. Questions were developed to be as precise and as unambiguous as possible with multiple choices and close-ended questions for accuracy. The questionnaire was divided into five sections with questions regarding: 1) demographics (including age, gender and nationality), 2) sources and handling of food and water during Hajj, 3) storage of food during Hajj, 4) prevention of FWBDs, and 5) GI symptoms and their management during the Hajj. Questions for the knowledge (4 questions) and practice (6 questions) sections of the questionnaire included elements form the WHO Five Keys to Safer Food [14]. The questionnaire’s content was reviewed by two public health experts with Hajj experience and face-validated by piloting it among 26 pilgrims before the study with feedback for doubtful or confusing items. The Cronbach’s alpha for the knowledge and practice sections of the questionnaire were 0.76, and 0.88, respectively, and were deemed acceptable.

A scoring system was used to score the knowledge and practice responses as described previously [15]. Briefly, incorrect or uncertain responses were given a 0 score, while a score of 1 was given for choosing the correct answer; a correct response was based on current literature and best practice. Overall mean scores, ranging from 0 to 1, for the knowledge and practice sections were calculated and were then further divided into four categories to reflect the level of knowledge and practice among pilgrims. These were: poor (score < 0.25), below average (score ≥ 0.25- < 0.50), above average (score ≥ 0.5- < 0.75), and good (score ≥ 0.75).

### Statistical analysis

Descriptive statistics such as mean and standard deviation (SD) were computed for quantitative variables and frequencies and percentages were calculated for categorical variables. The difference of knowledge and practice scores in relation to individual covariates was evaluated by the Mann–Whitney U test or Kruskal-Wallis test as appropriate. Binary logistic regression was used to identified independent predictors for having good knowledge (score ≥ 0.75) and good practice (score ≥ 0.75) in relation to food and water safety. Odds ratios with 95% CIs were computed to assess the presence and degree of association between the dependent versus independent variables. Variables having *p*-value < 0.05 at the bi-variable analysis were taken for multivariate analysis. Correlation between knowledge and practice was examined using the Spearman correlation coefficient. All of the tests for significance were two-sided and *p* values < 0.05 were considered statistically significant. All analyses were performed using SPSS 22.0 (SPSS Inc., Chicago, USA) software program.

### Ethics and confidentiality

All study participants were briefed about the study by trained researchers and gave verbal consent before enrolment. The study was approved by the King Fahad Medical City Ethics Committee and the Institutional Review Board (IRB log: 19-417E). The survey forms were anonymous and did not include any identifiers or personal information of the participants.

## Results

### Demographics of the study population

A total of 1363 pilgrims from 28 countries were enrolled in the study. Characteristics of the study population are summarized in Table 1. The mean age of participants was 50.1 years (SD = 12.3, range: 18–98 years) with over half (54.0%, *n* = 709) aged 46–65 years. Most participants were male (63.4%, *n* = 845) and originated mainly from Indonesia (16.2%, *n* = 215), Nigeria (13.8%, *n* = 183), Pakistan (10.5%, *n* = 139) and India (10.0%, *n* = 133).

**Table 1:** Demographics and gastrointestinal symptoms and their managements among the study population

Variable	Category	n	%
**Gender**		**1332**	
	Male	845	63.4
	Female	487	36.6
**Age**		**1313**	
	≤25	34	2.6
	26–45	440	33.5
	46–65	709	54.0
	< 65	130	9.9
**Nationality**		**1325**	
	Africa	432	32.6
	Asia	622	46.9
	Middle east	232	17.5
	Others^a^	39	2.9
**Experienced GI symptoms**	**Yes**	**133**	**9.7**
	Vomiting	28	21.1
	Nausea	27	20.3
	Abdominal pain	41	30.8
	Constipation	23	17.3
	Diarrhea	69	51.9
	Mucus or blood in stool	3	2.3
**Sought medical assistance**	**Yes**	**104**	**78.2**
	Medical mission doctor	77	74.0
	Hospital	12	11.5
	Primary health center	15	14.4
	Pharmacist	7	6.7
**Medication taken**	**Yes**	**103**	**77.4**
	Pain killer	49	47.5
	Anti-diarrhea	50	48.5
	Anti-constipation	14	13.6
	Antibiotic	45	43.7

*GI* gastrointestinal

^a^Europe, USA, Australia and New Zealand

### Gastrointestinal symptoms among pilgrims

Nearly 10% (*n* = 133) of pilgrims reported suffering at least one GI symptom during the Hajj (Table 1). Diarrhea was the most common symptom, reported by over half of pilgrims with GI symptoms, followed by nausea/vomiting (41.4%, *n* = 55), abdominal pain (30.8%, *n* = 41) and constipation (17.3%, *n* = 23). For the majority of pilgrims these symptoms lasted < 2 days. Most (78.2%, *n* = 104) of those with symptoms sought medical assistance (mainly from their medical mission doctor) and took medications for their ailment (mainly anti-diarrhea medications, pain killers and antibiotics) (Table 1).

### Sources of food and water during hajj

Nearly 30% (*n* = 393) of respondents reported bringing food to KSA from their country of origin, including raw and cooked food (Table 2). Most pilgrims (> 86%) declared that their main food source during their Hajj, including during the Hajj rituals days, was their hotel/Hajj mission. This was followed by restaurants and food outlets (≈45%) and food trucks and kiosks (≈12%). A small proportion of pilgrims (< 9%) reported cooking their own food during Hajj or buying it from street vendors. Most respondents (99.4%, *n* = 1324) drunk bottled water during the pilgrimage (Table 2).

**Table 2:** Food and water sources during Hajj

Variable	Category	n	%
**Brought food to KSA from country of origin**	**Yes**	**393**	**29.9**
	Raw food^a^	109	27.7
	Canned food	148	37.6
	Cooked food	107	27.2
**Sources of food during Hajj (non-ritual days)**	Cooked own food	115	8.7
	Hotel/Hajj mission	1149	86.8
	Restaurant/food outlet	648	48.9
	Street food truck/kiosk	159	12.0
	Street vendor	84	6.3
**Sources of food during Hajj (ritual days)**	Cooked own food	120	9.0
	Hotel/Hajj mission	1216	91.6
	Restaurant/food outlet	582	43.9
	Street food truck/kiosk	168	12.6
	Street vendor	99	7.4
**Water sources during Hajj**	Tap water	38	2.8
	Water bottle	1324	99.4
	Street water points	415	31.1

*KSA* Kingdom of Saudi Arabia

^a^including fruits and vegetables

### Food and water-related practices among pilgrims

Outside the Hajj setting, the majority of pilgrims separated kitchenware for raw and cooked food (80.2%, *n* = 1036), did not consume food past its expiry date (95.4%, *n* = 1261), cleaned food preparation surfaces before use (92.2%, *n* = 1198) and washed fruits and vegetables before consumption (90.9%, *n* = 1187). Also, most pilgrims (> 92%) washed their hands before and after cooking and eating, and after using the bathroom (Table 3). However, only 43.1% (*n* = 561) reported that they do not thaw frozen food at room temperature.

**Table 3:** Food and water-related practices among pilgrims

Practice	Category	n	%
**General practice**			
**Use different kitchenware for raw and cooked food or wash them thoroughly between use**		**1292**	
	Yes	1036	80.2
	No	171	13.2
	Sometimes	85	6.6
**Ate food past its expiry date**		**1323**	
	Yes	31	2.3
	No	1261	95.4
	Not sure	31	2.3
**Thaw frozen food at room temperature**		**1301**	
	Yes	614	47.2
	No	561	43.1
	Sometimes	126	9.7
**Clean food preparation surfaces before use**		**1300**	
	Yes	1198	92.2
	No	16	1.2
	Sometimes	86	6.6
**Wash fruits/vegetables before consumption**		**1306**	
	Yes	1187	90.9
	No	17	1.3
	Sometimes	102	7.8
**Wash hands**	Before eating	1219	92.1
	After eating	1307	98.2
	Before cooking	1122	90.1
	After cooking	1185	95.0
	Before using the toilet	710	55.0
	After using the toilet	1299	98.4
**Practice during Hajj**			
**Carried food while performing Hajj rituals**	**Yes**	**373**	**30.3**
	Raw food^a^	239	64.1
	Canned food	119	31.9
	Cooked food	32	8.6
**Stored cooked for > 2 h during Hajj**	**Yes**	**277**	**21.0**
	In a fridge	157	56.6
	At room temperature	146	52.7
**Eaten cooked food stored at room temp for > 2 h**	**Yes**	**231**	**17.4**
**Shared water/liquids with others**	**Yes**	**218**	**16.3**
	From the same cup	32	14.6
	From the same bottle	186	85.4
**Shared food with others**	**Yes**	**339**	**25.4**
	Using the same utensils	30	4.0
	Using the same plate	308	40.8
**Persons shared food and water with**			
	Family	533	95.7
	Friends	343	61.6
	Roommates/Hajj mission	229	41,1
	Other Hajjis	66	11.8

^a^including fruits and vegetables

During Hajj, 30.3% (*n* = 373) of pilgrims carried food with them while performing the Hajj rituals, mainly in the form of raw (e.g. fruits and vegetables) or canned food (Table 3). Also, 21% (*n* = 277) of respondents reported having stored cooked food for > 2 h, and over half of them (52.7%, *n* = 146) stored it at room temperature. Similarly, 17.4% (*n* = 231) of respondents did eat cooked food stored at room temperature for > 2 h. During the pilgrimage, 16.3% (*n* = 218) of pilgrims shared water/liquids with others (mainly from the same bottle) and 25.4% (*n* = 339) shared food. Food and water were mainly shared among family and friends, roommates or pilgrims from the same Hajj mission (Table 3).

### Food and water safety knowledge and practice among pilgrims

Food and water safety knowledge and practice among pilgrims were assessed using four knowledge questions and six practice statements. The mean scores for each knowledge and practice statement are presented in Table 4. Most pilgrims (> 78%) were aware that raw and cooked food should be separated both during food preparation and during storage. Also, most (> 75%) recognized that some food should not be stored at room temperature and that once thawed, frozen raw food should not be frozen again for future consumption.

**Table 4:** Food and water safety knowledge and practice scores for pilgrims

Statement	N	min	max	mean	SD
**Knowledge**					
Cooked and raw food should be prepared with separate equipment.	1363	0	1	0.78	0.41
Cooked and raw food should be stored separately	1299	0	1	0.85	0.36
All food can be stored at room temperature	1327	0	1	0.78	0.41
Frozen raw food (e.g. meat, fish, chicken) can be frozen again after being thawed for later consumption	1311	0	1	0.75	0.43
**Overall knowledge score**	**1363**	**0**	**1**	**0.77**	**0.28**
**Practice**					
Use different kitchenware for raw and cooked food or wash them thoroughly between use	1290	0	1	0.80	0.39
Ate food past its expiry date	1323	0	1	0.95	0.21
Thaw frozen food at room temperature	1301	0	1	0.43	0.49
Ate cooked food stored at room temp for > 2 h	1329	0	1	0.78	0.41
Clean food preparation surfaces before use	1300	0	1	0.92	0.26
Wash fruits/vegetables before consumption	1306	0	1	0.91	0.28
**Overall practice score**	**1363**	**0**	**1**	**0.77**	**0.20**

*Min* minimum, *Max* maximum, *SD* standard deviation, *N* number of observations

According to our scale, pilgrims had a good level of knowledge and practice regarding food and water safety with overall mean scores of 0.77 (SD = 0.28) and 0.77 (SD = 0.20), respectively (Table 4). Nearly 72% (*n* = 983) of pilgrims had an overall mean knowledge score ≥ 0.75 (good knowledge) and 62.1% (*n* = 847) had an overall mean practice score ≥ 0.75 (good practice).

### Variables and knowledge and practice scores

There was a statistically significant difference in knowledge and practice scores in relation to gender, nationality and experiencing GI symptoms but not age (Table 5). Females had higher knowledge and practice scores than males. Pilgrims from Europe, USA, Australia and New Zealand had the highest knowledge and practice scores. Also, pilgrims who suffered GI symptoms during Hajj had lower knowledge and practice scores compared to those who did not report symptoms.

**Table 5:** Variables and food and water safety knowledge and practice scores

Variable		N	Knowledge score			Practice score		
			Median	IQR	***p***-value	Median	IQR	***p***-value
**Gender**	Male	845	0.75	0.50	**< 0.0001**	0.83	0.17	**0.011**
	Female	487	1.00	0.25		0.83	0.17	
**Age**	≤25	34	0.75	0.50	0.055	0.66	0.17	0.085
	26–45	440	0.75	0.25		0.83	0.17	
	46–65	709	1.00	0.50		0.83	0.17	
	< 65	130	0.75	0.50		0.83	0.17	
**Nationality**	African	432	0.75	0.25	**< 0.0001**	0.83	0.33	**< 0.0001**
	Asian	622	0.75	0.50		0.83	0.17	
	Middle East	232	1.00	0.25		0.83	0.17	
	Others^a^	39	1.00	0.25		1.00	0.17	
**GI symptoms**	No	1230	0.75	0.50	**0.005**	0.83	0.17	**0.006**
	Yes	133	0.75	0.50		0.83	0.33	

*p*-value for the Mann–Whitney U or Kruskal-Wallis test

*N* number of observations, *SD* standard deviation, *IQR* interquartile range, *GI* gastrointestinal

^a^Europe, USA, Australia and New Zealand

Multivariate analysis identified several independent predictors for having good knowledge and practice in relation to food and water safety (Table 6). Gender, nationality and suffering GI symptoms during Hajj were significantly associated with good knowledge and practice. The odds of having good knowledge or good practice were ≈ 40% lower in males compared to females (adOR = 0.63 [95% CI = 0.48–0.83] and 0.59 [95% CI = 0.46–0.75], respectively). Compared to pilgrims from Africa, pilgrims from Asia had 40% lower odds of having good knowledge (adOR = 0.60; 95% CI = 0.45–0.79) and pilgrims from Europe, USA, Australia and New Zealand were 11 times more likely to exhibit good practice (adOR = 10.9; 95% CI = 2.58–46.5). Pilgrims who suffered GI symptoms during their Hajj were less likely to have good knowledge (adOR = 0.55; 95% CI = 0.37–0.81) or exhibit good practice (adOR = 0.65; 95% CI = 0.44–0.95) compared to those who did not report symptoms.

**Table 6:** Association between variables and good knowledge and practice

Variable	N	Good Knowledge		Good Practice	
		OR [95%]	aOR [95%]	OR [95%]	aOR [95%]
**Gender**					
Female	845	1	1	1	1
Male	487	0.64 (0.49–0.83)^a^	0.63 (0.48–0.83)^a^	0.61 (0.48–0.77)^a^	0.59 (0.46–0.75)^a^
**Age (years)**					
≤ 25	34	1	–	1	–
26–45	440	1.92 (0.93–3.97)	–	1.95 (0.96–3.92)	–
46–65	709	1.74 (0.85–3.54)	–	2.06 (1.03–4.12)^a^	–
< 65	130	1.09 (0.50–2.38)	–	1.48 (0.69–3.17)	–
**Nationality**					
African	432	1	1	1	1
Asian	622	0.62 (0.47–0.82)^a^	0.60 (0.45–0.79)^a^	0.94 (0.73–1.21)	0.97 (0.75–1.26)
Middle east	232	1.48 (0.98–2.22)	1.42 (0.94–2.15)	1.02 (0.73–1.43)	1.03 (0.73–1.46)
Others^b^	39	1.41 (0.60–3.29)	1.28 (0.54–3.01)	5.19 (1.81–14.8)^a^	10.9 (2.58–46.5)^a^
**GI symptoms**					
No	1230	1	1	1	1
Yes	133	0.58 (0.40–0.85)^a^	0.55 (0.37–0.81)^a^	0.64 (0.45–0.92)^a^	0.65 (0.44–0.95)^a^

*OR* odds ratio, *aOR* adjusted odds ratio, *GI* gastrointestinal

^a^statistically significant

^b^Europe, USA, Australia and New Zealand

There was a moderate but statistically significant positive correlation between knowledge and practice scores (r_s_ = 0.41, *p* < 0.0001). The correlation was not significantly affected by age, gender or nationality.

## Discussion

The Hajj mass gathering is a risk for FWBDs and outbreaks [9–11]. Crowded conditions at the event, shared accommodations and hot weather are some of the contributing factors. Knowledge of and adherence to food and water safety measures by pilgrims are paramount to reduce the risk of FWBDs at the event. The current study investigated Hajj pilgrims’ knowledge and practice concerning food and water safety and documented the prevalence of GI symptoms among this population. Nearly 10% of pilgrims suffered GI symptoms during Hajj, especially diarrhea. Overall, pilgrims had good knowledge and practice regarding food and water safety and knowledge moderately correlated with practice. Gender, nationality and suffering GI symptoms during Hajj were significantly associated with good knowledge and practice.

A systematic review conducted on literature published between 1980 and 2016 found that at least 15 outbreaks of gastrointestinal infections occurred in the context of mass gatherings outside Hajj and Umrah with an estimated incidence of 9- > 55,000 per 100,000 attendees [16]. Overall, the estimated incidence of gastrointestinal diseases per 100,000 attendees ranged from 9 to > 55,000 during these outbreaks. These outbreaks were caused by a variety of organisms including bacteria (e.g. *Escherichia, Shigella*, and *Salmonella*), viruses (e.g. norovirus and hepatitis A) and parasites (e.g. *Cryptosporidium*) and were the results of non-compliance with hygiene rules and inadequate sanitation [16]. In the Hajj context, despite improvements in services, food safety and waste management and hygiene standards, GI diseases, food-poisoning outbreaks, and diarrhea continue to occur among pilgrims [10, 17]. A study among 376 French pilgrims between 2016 and 2018 reported that 18.6% presented at least one GI symptom during Hajj and 13.8% had diarrhea [18]. A review of hospital data between 2002 and 2010 found a mean prevalence of GI disease among ill pilgrims of 12.4% (range: 2.4–17.4%) [10]. The same study reported that among 262,999 pilgrims surveyed over 12 years (2002–2013), the mean diarrhea prevalence was 2.3% (range: 1.1–23.3%). Our results are within the above ranges, where at least 9.7% of pilgrims we surveyed experience GI symptoms and 5.1% suffered diarrhea. The etiology of GI disease in Hajj is not well characterized. However, a large-scale study among pilgrims with diarrheal infection during the 2011–2013 Hajj seasons found the main agents were bacteria (82.9%), followed by viruses (6.1%) and parasites (5.3%) [19]. *Salmonella* spp., *Shigella*/enteroinvasive *E. coli*, and enterotoxigenic *E. coli* were the main pathogens associated with severe symptoms. The Hajj also seems to favor acquisition of enteric pathogens [18, 20]. For example, one report found that 36.4% of French pilgrims attending the 2016–2018 Hajj had acquired at least one enteric pathogen [18]. Enteropathogenic and Enteroaggregative *E. coli* were the most frequently acquired pathogens by pilgrims (17.6 and 14.4%, respectively).

The study found that 30% of pilgrims brought food from their home country, which is lower than reported in earlier studies; 39% in 1997, 37% in 1998 and 35% in 1999 [21–23]. Most pilgrims (> 86%) obtained their food from their hotel/Hajj mission during their pilgrimage and only a minority (< 7.4%) consumed food from street vendors. This is in accordance with the observed trend in pilgrims relying more on food provided by their Hajj missions and less on food bought from street vendors. For example, in 1997–1999, 27–43% of pilgrims in Mina obtained food from street vendors [21–23], while in 2011, only 11.3% of pilgrims did so [24]. Similarly, the prevalence of pilgrims obtaining food from their Hajj mission increased from 54% in 1997, to 61% in 2002 to 79% in 2007 [25]. Hajj missions are responsible for providing and supervising safe food services in Hajj especially during the Hajj ritual days [26]. This includes supervising cleanliness of the cooking and dining areas and supervision of the served food. Therefore, the risk of FWBDs is lower if pilgrims obtain food through their Hajj mission’s services rather than other sources such as street vendors and food establishments which may not strictly adhere to food safety regulations [27].

Nearly all pilgrims in our study (99.4%) drunk bottled water, which is a safer source of water. A smaller proportion (31.1%) also used street water points and few (2.8%) used tap water. This reflects the increasing trend among pilgrims of using bottled water and safer water sources in general. In 2002, 54% of pilgrims in Mina reported drinking water from water bottles, 49% from coolers available in the camp, 7% from tankers, 5% from plastic bags, 3% from taps and 2% from other sources [28]. In 2007, 25% used water coolers or water tanks and 73% used plastic bottles and plastic bags [25]. Improvements of services provided for pilgrims including in their camps during the Hajj rituals and availability of free water bottles, are thought to have contributed to the decline in use of risky water sources at the pilgrimage [24].

In general, most pilgrims had good knowledge and self-reported practice in relation to self-hygiene and keeping food preparation areas clean, separating raw and cooked food and use of safe water and raw materials. However, some hazardous practices were noted in relation to not keeping food at safe temperatures (thawing frozen food at room temperature and storing cooked food at room temperature for > 2 h or consuming such food). Food and water safety studies from both developed and developing countries report that the proportions of consumers with adequate knowledge and self-reported practice regarding good hygiene (e.g. hand washing), prevention of cross contamination (e.g. separating raw and cooked food) and ensuring safe raw materials (e.g. washing fruits and vegetables and not using food beyond expiry date) are generally high [5, 6, 29]. Although these proportions varied across studies and did not always reflect actual practice [5, 6]. However, similar to our results, these studies found poor practice in relation to proper defrosting and storage of food. In one meta-analysis, only 51–67% of consumers practiced proper defrosting and kept food at safe temperatures [5]. As reported in previous Hajj studies [24, 25, 28], we also noted some other risky behavior among pilgrims such as unsafe sharing of food and water and carrying food with them during the Hajj rituals. Such behaviors may be born out of the feelings of unity and selflessness that Hajj inspires among pilgrims or out of necessity given that the Hajj rituals take place at different locations.

Studies reported considerable differences in food and water safety knowledge, attitude and behavior across demographic, socioeconomic and cultural variables including age, gender, ethnicity, occupation, levels of income and education as well as geographical location [5, 29, 30]. As was the case in our study; other investigators reported that in general, females tend to have significantly better knowledge, attitude and practice regarding food and water safety compared to males [5, 29]. However, contrary to our findings, an international survey among consumers from Africa and Asia found that respondents from Asia had better food safety knowledge and practice than those from Africa [29]. This is likely due to the difference in the countries included in both studies. The study also found that pilgrims who suffered GI symptoms during Hajj were less likely to have good knowledge and practice regarding food and water safety. Lack of knowledge and risky behavior regarding water and food handling, storage and preparation can increase the likelihood of FWBDs. In one study, knowledge attitude and practice regarding FWBDs of communities affected by a large diarrhea outbreak was compared to that of communities not affected by the outbreak [30]. The mean knowledge attitude and practice scores were higher among the non-affected populations compared to those affected by the outbreak. Scores were low among those who had severe diarrhea in the previous 30 days. Authors hypothesized that poor knowledge, negative attitude and weak practice among these individuals could have rendered them more susceptible to FWBDs [30]. Our results support such assumption.

Similar to others [29], we found a moderate but statistically significant positive correlation between food and water safety knowledge and practice scores. This reflects the fact that there is no simple relationship between the level of knowledge, attitude and behaviors and that good knowledge of food and water safety does not always translate to good practice [5, 6]. In fact, food safety studies indicate no strong correlation between knowledge, attitudes, behavioral intentions, self-reported practices and observed food safety behaviors among consumers [6]. In one UK study, although 100% of participants correctly answered knowledge question regarding hand washing when handling food and separating raw and ready-to-eat foods, only 45–80% reported always implementing the correct practice and even fewer (0–48%) actually performed the correct practice at all times during the observed food preparation [31]. Such discrepancy between knowledge and practice was also reported in Hajj. For example, Gautret et al. [32] found that although 13.9% of French pilgrims surveyed knew that drinking raw camel milk could cause diseases, 40.6% would drink it if it were given during the Hajj. Not following safe practices despite adequate knowledge of food and water safety may be due to many reasons including a negligent attitude or lack of concern, underestimating the risk and optimistic bias wherein participants perceive that negative effects are relatively unlikely, or voluntary acceptance of the risk [5, 6].

Our study has some limitations. While we enrolled a large number of pilgrims from different countries, the sample size represents a small proportion of the overall Hajj population at large. The latter, in addition to the potential for volunteer bias, limits the generalizability of the findings. Also, data were collected using a questionnaire; therefore, responses obtained were prone to information bias and there was a small proportion of missing data. Similarly, we did not observe practice; hence, the results may not accurately reflect actual practice among pilgrims. Studies that relied on self-reporting of behaviors found evidence of participants’ tendency to over report behaviors perceived to be “good” (social desirability bias) [33, 34].

In summary, this large cross-sectional study found that up to 10% of pilgrims suffered from GI symptoms during Hajj. Most pilgrims had good knowledge and good self-reported practice in relation to food and water safety and results show improvement from previous studies among Hajj pilgrims. These findings may reflect the significant improvements in food safety and waste management, hygiene standards and services provided in Hajj as well as the strict regulations regarding the importation of food during the pilgrimage and enhancement in risk communication and health proportion targeted at pilgrims and their Hajj missions. While our results are encouraging, we did note several risky behaviors among pilgrims, both general and Hajj-specific. While these were often among small proportions of pilgrims, it is worth noting that such proportions can translate to a sizable number of pilgrims on a Hajj population scale. These unsafe practices may render many pilgrims at risk of FWBDs and may lead to outbreaks during the event. Continuous risk communication, training and educational interventions are needed to improve pilgrims’ knowledge and behavior concerning food and water safety. Such interventions have been shown to have a positive impact on the knowledge and practice of both consumers and food handlers [35, 36]. However, the acquisition of knowledge alone does not automatically produce the corresponding behavior, nor will it necessarily lead to appropriate changes in behavior [6]. Additional research is needed to identify reasons other than knowledge that drive the safe behavior of pilgrims, which can help develop appropriate, targeted and tailored health promotion and risk communication programs and materials. Most outbreak of GI disease at mass gatherings are non-vaccine preventable [16], hence preventive measures for safe food and water need to be followed by pilgrims to reduce the burden of these illnesses at the Hajj. Ultimately, preventing FWBDs is not the responsibility of a single entity, but a shared obligation of producer, processors, regulators as well as consumers [3].

## Data Availability

The datasets used and analysed during the current study are available from the corresponding author on reasonable request.
